# Properties of a Newly Identified Esterase from *Bacillus* sp. K91 and Its Novel Function in Diisobutyl Phthalate Degradation

**DOI:** 10.1371/journal.pone.0119216

**Published:** 2015-03-06

**Authors:** Junmei Ding, Chaofan Wang, Zhenrong Xie, Junjun Li, Yunjuan Yang, Yuelin Mu, Xianghua Tang, Bo Xu, Junpei Zhou, Zunxi Huang

**Affiliations:** 1 Engineering Research Center of Sustainable Development and Utilization of Biomass Energy, Ministry of Education, Yunnan Normal University, Kunming 650500, Yunnan, China; 2 Key Laboratory of Enzyme Engineering, Yunnan Normal University, Kunming 650500, Yunnan, China; Montana State University, UNITED STATES

## Abstract

The widely used plasticizer phthalate esters (PAEs) have become a public concern because of their effects on environmental contamination and toxicity on mammals. However, the biodegradation of PAEs, especially diisobutyl phthalate (DiBP), remains poorly understood. In particular, genes involved in the hydrolysis of these compounds were not conclusively identified. In this study, the *CarEW* gene, which encodes an enzyme that is capable of hydrolyzing ρ-nitrophenyl esters of fatty acids, was cloned from a thermophilic bacterium *Bacillus* sp. K91 and heterologously expressed in *Escherichia coli* BL21 using the *pEASY*-E2 expression system. The enzyme showed a monomeric structure with a molecular mass of approximately 53.76 kDa and pI of 4.88. The enzyme exhibited maximal activity at pH 7.5 and 45°C, with ρ-NP butyrate as the best substrate. The enzyme was fairly stable within the pH range from 7.0 to 8.5. High-pressure liquid chromatography (HPLC) and electrospray ionization mass spectrometry (ESI-MS) were employed to detect the catabolic pathway of DiBP. Two intermediate products were identified, and a potential biodegradation pathway was proposed. Altogether, our findings present a novel DiBP degradation enzyme and indicate that the purified enzyme may be a promising candidate for DiBP detoxification and for environmental protection.

## Introduction

Phthalate esters (PAEs) are important synthetic organic compounds not only applied as plasticizers (softeners) on a massive scale in the industrial production of plastics, but also used as additives in the manufacture of adhesives, paints, lacquers, cardboard, cosmetics, and so on [1]. To provide the required flexibility, PAE plasticizers are not bound covalently to the plastic matrix and are thus capable of leaching into the environment [2]. Therefore, PAEs have been widely detected in both aquatic and terrestrial environments [3]. After a half century of supposedly safe use, the impacts of PAEs on ecological systems and human health raised serious concerns because of the adverse effects on animal cells. PAEs can bind to estrogen receptors and disrupt the endocrine system [4]. These compounds can induce reproductive toxicity [5], hormonal disorders [6], hepatic peroxisome proliferation [7], hepatocellular tumors [8], and may harm fetal health [9]. This increased understanding has fundamentally changed the perception on the environmental and health risks presented by PAEs and have raised an important issue on their environmental fate.

Biodegradation refers to the chemical decomposition of organic substances by living organisms or other biological means, and microbial degradation is suggested to be the principal mechanism for removing PAEs from the environment [10]. During the past 40 years, biochemical pathways, kinetics, and related molecular mechanisms of microorganisms in the degradation of PAEs have been extensively investigated. The biodegradation of PAEs was first detected in river water and acclimated activated sludge [11]. Thereafter, several microorganisms, such as *Pseudomonas* species [12,13], *Pelotomaculum* and *Desulfotomaculum* species [10], *Gordonia* sp. strain MTCC 4818 [14], *Micrococcus* sp. YGJ1 [15], and a number of uncharacterized microorganisms in sludge capable of degrading PAEs pollutants in the environment, have been identified [16,17]. The first step in the bacterial degradation of PAEs is initiated by de-esterification reactions to form a monoalkyl phthalate and the corresponding alcohol, with the involvement of esterases as key enzymes [18]. A second ester bond is then hydrolyzed, forming phthalic acid (PTH) that can be completely mineralized via protocatechuate formation by aerobic microorganisms [19–22]. However, the biochemistry of the first PAEs cleavage step remains poorly understood. As of this writing, only a few enzymes involved in this reaction have been reported; these enzymes include dimethyl terephthalate (DMT) esterase from *Fusarium* sp. DMT-5-3 [23], two distinct PAE hydrolases in *Micrococcus* sp. YGJ1 [15,24], mono-2-ethylhexyl phthalate hydrolase from *Gordonia* sp. P8219 [25], ester hydrolase PatE from *Rhodococcus jostii* RHA1 [26], dibutyl phthalate (DBP) hydrolase from the wastewater treatment plant metagenomic library [27], DBP hydrolase from *Acinetobacter* sp. M673 [28], and esterase EstS1 from *Sulfobacillus acidophilus* DSM10332 [29]. Only the genes and proteins of the latter five enzymes have been identified. Two more hydrolases, porcine and bovine pancreatic cholesterol esterases [30], and *Fusarium oxysporum* f. sp. pisi cutinase [31] were also found active in PAEs degradation. However, all the enzymes referred above have no more than 50% sequence identities with enzyme CarEW (S1 Table).

DiBP is one of the main plasticizers in PAEs, and it has some similarities to DBP. DiBP has adverse effects to human health [32]. However, the biodegradation of DiBP has not been reported to date. In the present study, we described a mesophilic enzyme from a thermophilic bacterium, *Bacillus* sp. K91 isolated from a hot spring water. This enzyme displayed specific hydrolase activity toward DiBP. The experimental data from electrospray ionization mass spectrometry (ESI-MS) and high-pressure liquid chromatography (HPLC) led us to propose a potential catabolic pathway for the degradation of DiBP. To our knowledge, we are the first to provide the combined genetic and enzymological characterization of a DiBP hydrolase that has a potential role for bioremediation.

## Materials and Methods

### Ethics statement

No specific permits were required for the described field studies. The field studies did not involve any endangered or protected species. The strain in this study was collected from the “Large Roll Pan hot spring” in Tengchong Volcano Geothermal National Geological Park (E 98° 23'–98° 39', N 24° 53'–25° 27'), Yunnan Province, China, with the permission of the authorities of the Tengchong Volcano Geothermal National Geological Park.

### Strains and reagents

*E*. *coli* BL21 was from Novagen (Merck). DiBP (99% purity) was from J&K Scientific Ltd., China. The standard sample PTH (99%) was from Beijing Haianhongmeng Reference Material Technology Co., Ltd., China. MiBP was from Shanghai PheroWeCan Co.,Ltd (99% purity). Nickel-NTA agarose was purchased from Qiagen (Germany). TransGen Biotech (Beijing) provided fast *pfu* DNA polymerase and *pEASY*-E2 expression kit. Different substrates, such as ρ-NP acetate (ρ-NPC_2_), ρ-NP butyrate (ρ-NPC_4_), ρ-NP caproate (ρ-NPC_6_), ρ-NP caprylate (ρ-NPC_8_), ρ-NP caprate (ρ-NPC_10_), ρ-NP laurate (ρ-NPC_12_), ρ-NP myristate (ρ-NPC_14_), and ρ-NP palmitate (ρ-NPC_16_), were from Sigma-Aldrich (USA) or TCI (Tokyo, Japan). Genomic DNA isolation, DNA purification, and plasmid isolation kits were from TianGen (China). All other chemicals were analytical grade.

### Genome sequencing and sequence analysis

Genomic DNA of *Bacillus* sp. K91 was extracted using a TianGen genomic DNA isolation kit from cells grown overnight at 50°C. Genome sequencing was performed by Beijing Genomics Institute (Guangzhou, China) using a Solexa Genome Analyzer, and a partial genomic sequence was obtained. Oligonucleotide primers were synthesized by Shanghai Sangon Biological Engineering Technology and Services Co., Ltd. (Shanghai, China). The full-length carboxylesterase gene *CarEW* was revealed based on the prediction of ORFs from the partial genomic sequence by the GeneMark.hmm online tool (version 2.8; http://exon.gatech.edu/GeneMark/gmhmm2_prok.cgi). Putative functions were inferred using the Basic Local Alignment Search Tool (BLAST) (http://www.ncbi.nlm.nih.gov/BLAST). Protein similarity search and alignment were performed using the data from CLUSTAL W [33]. The signal sequence for peptide cleavage in the amino acid sequences of CarEW was predicted using SignalP 4.0 [34]. The Pfam database (version 27.0, available at http://pfam.sanger.ac.uk/) were used to search for conserved domains in the predicted amino acid sequences. ESPript output was used to render the analysis of multiple sequence alignment [35]. The neighbor-joining method in the molecular evolutionary genetic analysis software package MEGA (version 6.0) was used to construct a phylogenetic tree. The theoretical molecular mass and isoelectric point of the deduced CarEW protein sequence was calculated using the Compute pI/Mw tool on the ExPASy proteomics server (available at http://expasy.org/tools/pitool.html).

### Expression and purification of recombinant CarEW

The *CarEW* gene was amplified using primers P1 (forward): 5’-ACTCATCAAATAGTAACGAC-3’ and P1 (reverse): 5’-TTCTCCTTTT GAAGGGAATAG-3’. Initial activation of the *Taq* DNA polymerase was performed for 5 min at 94°C, followed by 35 cycles as follows: 94°C for 30 s, then 58°C for 30 s, 72°C for 2 min, followed by a final extension at 72°C for 10 min and holding of samples at 4°C. After PCR, the products were verified by electrophoresis on a 1.2% agarose gel using a 1 kb ladder.

The expression of *CarEW* gene was then undertaken using the *pEASY*-E2 expression kit following the manufacturer’s instructions. The PCR product was ligated to the *pEASY*-E2 vector and introduced into *E*. *coli* BL21 cells. Twenty colonies were picked for secondary screening and their insert was analyzed for size and orientation by colony PCR using the gene specific forward primer and the vector specific *pEASY*-E2 T7 terminator primer (T7 ter: CCACCGCTGAGCAATAACTA). Protein expression was accomplished by growing and inducing 50 mL of cells as follows: 5 mL of Luria-Bertani (LB) broth containing 100 μg∙mL^−1^ ampicillin were inoculated with a single colony and grown overnight at 37°C with shaking. Subsequently, 50 mL of LB broth containing 100 μg∙mL^−1^ of ampicillin was inoculated with 1 mL of the overnight culture and grown until OD_600_ reached 0.6. The culture was then induced with 0.7 mM IPTG and grown at 20°C with shaking at 160 rpm for 20 h. The cells were then harvested by centrifugation at 12,000 rpm at 4°C for 20 min, and the pellets were stored at −80°C before proceeding to protein purification. Purification of the proteins was carried out in native conditions using the ProBond Purification System (Invitrogen, USA). Success of the purification was examined using sodium dodecyl sulfate-polyacrylamide gel electrophoresis (SDS–PAGE). Protein concentration was estimated using the Bradford procedure employing BSA as the standard (Sigma) [36].

### Assay of Enzyme activity

Enzyme activity was quantified on a microplate reader (Bio-Rad, USA) based on the level of ρ-nitrophenol (ρ-NP) released following the hydrolysis of ρ-NP ester substrates by the enzyme [37]. The production of ρ-NP was monitored in triplicate every minute for 5 min at 405 nm. Unless otherwise described, enzyme activity was measured by a standard assay at 45°C, with 0.6 mM ρ-NP ester substrates in 50 mM phosphate buffer (pH 7.5), 0.36% Triton X-100, 0.1% gum Arabic, and 51 μg of the purified CarEW. The substrate used in standard conditions was ρ-NPC_4_ for CarEW. Blank reactions were performed with every measurement to subtract appropriate values for non-enzymatic hydrolysis of the substrate. One unit enzyme activity (U) was defined as the amount of activity required to release 1 μmol of ρ-NP per min from ρ-NP ester under assay conditions. Michaelis-Menten kinetics of different substrates catalyzed with EstD1 were identified by plotting reaction rates against various concentration of substrates (ρ-NPC_2_, ρ-NPC_4_, ρ-NPC_6_, ρ-NPC_8_, ρ-NPC_10_, ρ-NPC_12_, ρ-NPC_14_, and ρ-NPC_16_) ranging from 0.1–1.2 mM from three independent sets of experiments. The ρ-NP ester substrates with C_2_ to C_16_ acyl chains were dissolved in acetonitrile at a concentration of 10 mM. For determination of the kinetic parameters for DiBP and MiBP, both were dissolved in methanol firstly. DiBP and MiBP degradation by CarEW was determined by the method of Hara et al. with some modifications [26].The recombinant CarEW hydrolase assays were performed in 10 mM citric acid-Na_2_HPO_4_ solution (pH 7.5) with 10 mM of DiBP or MiBP esters. Kinetic parameters of each compound were collected at a concentration range of 0.1–1.2 mM. Each sample was incubated at 45°C with the enzyme at a final concentration of 10 U∙mL^−1^ (300 μg) for 120 min. The substrate amount was then determined by HPLC/MS analysis every 20 min for each reaction. At least three independent determinations were performed for each kinetic constant. The substrate-free assay system was also used as blank simultaneously. Kinetic values were calculated from nonlinear regression data analysis against various substrate concentrations using Grafit 7.0 software (R. J. Leatherbarrow, Erithicus Software, Ltd., Horley, United Kingdom).

### HPLC/MS analytical methods

A reaction mixture containing enzyme, DiBP or MiBP in 10 mM citric acid-Na_2_HPO_4_ solution (pH 7.5) was incubated at 45°C with the total reaction volume was 1 mL. After incubating the reaction mixtures for indicated time, the reaction was stopped by adding 10% (v/v) of 1 N HCl to the mixture, and the reaction products were extracted using the same volume of ethyl acetate. After dried over Na_2_SO_4_, the samples were re-dissolved in methanol. The residual and separated DiBP, MiBP and PTH were calculated based on the resulting peak areas by using an Agilent (Agilent 1100) spectrometry system. The operating conditions of the mobile phase 0.1% phosphorous acid solution/acetonitrile (10:90, v/v) were applied. The detector, wavelength, and flow rate were DAD, 230 nm, and 1 mL∙min^−1^, respectively. The column, temperature, and injection volume were Zorbax Eclipasse XDB-C18 (4.6 mm × 150 mm, 5 μm Agilent), 30°C and 3 μL, respectively. The chromatographical analysis was carried out on a Waters Acquity-Xevo TQ system (Waters, Milford, MA, USA) using positive and negative electrospray ionization (ESI) under the following conditions: capillary voltage was 2.0 kV, cone −30.0 V and 30 V, source temperature 350°C, desolvation temperature 200°C, desolvation gas flow 800 L∙h^−1^; and cone gas flow 150 L∙h^−1^. Full scan and daughter ions scan were used to monitor the analytes.

### Biochemical characterization of CarEW

Biochemical characterization of CarEW was examined by incubating purified CarEW with different temperatures, pH, metal ions, chemical agents, and organic solvents. The effect of pH on the purified enzyme was tested at 37°C, with the pH in range from 5.0 to 10.0 using the following buffers (50 mM): citrate phosphate buffer (pH 5.0 to 8.0), Tris-HCl (pH 8.0 to 9.0), and boric acid (pH 9.0 to 10.0). The pH stability of CarEW was also determined with the same buffers. The residual enzyme activity was measured after 1 h incubation at 37°C under standard assay. The optimum temperature for enzyme activity was determined at temperatures between 0°C to 75°C at the optimum pH (50 mM phosphate buffer) using ρ-NPC_4_ as the substrate. Thermostability of the enzyme was examined using ρ-NPC_4_ as substrate under standard assay after incubation of the enzyme for 1 h at 37, 45, and 55°C. The relative catalytic activity of the pre-incubated sample at 45°C and pH 7.5 was regarded as 100%.

The effect of different potential inhibitors or activators (metal ions, chemical agents, and organic solvents) on the enzyme activity was also determined using a standard assay with ρ-NPC_4_ as the substrate. The activity was measured at 45°C immediately after each compound was mixed with the enzyme and after 5 min of incubation. The activity assayed in the absence of inhibitors or activators was defined as the control.

### Nucleotide sequence accession numbers

The nucleotide sequences of the 16S rRNA and *CarEW* gene were deposited in the GenBank database under accession numbers KJ131181 and KM098150, respectively.

## Results and Discussions

### Sequence analysis of *CarEW*

**Fig 1:**
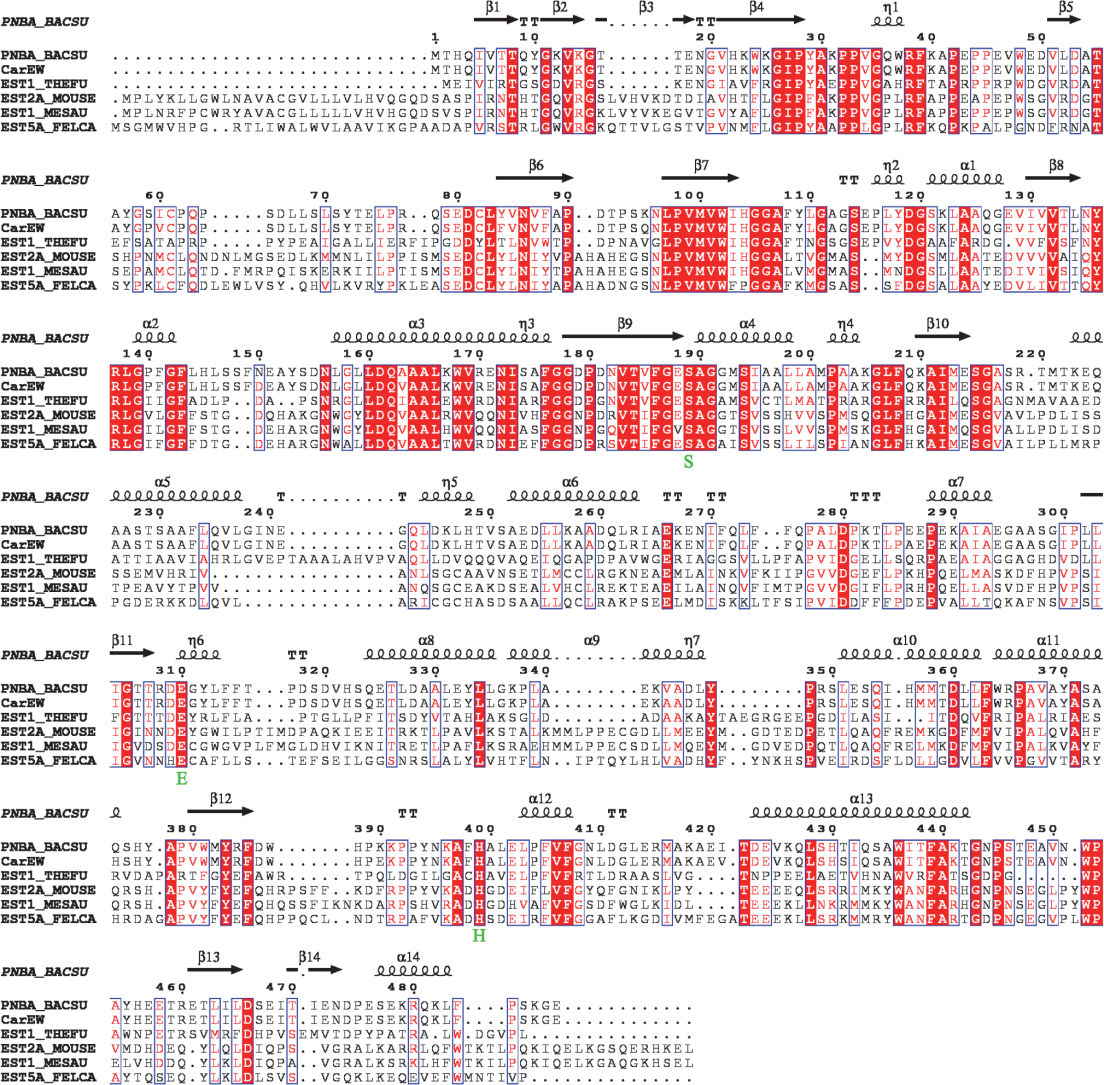
Protein sequences alignment between CarEW and homologs from the carboxylesterase family. ESPript outputs obtained with the sequences from the SWISSPROPT databank and alignment with CLUSTAL W. Sequences are grouped according to similarity. PNBA_BACSU, PNB (Para-nitrobenzyl esterase) carboxylesterase from *B*. *subtilis* subsp. Subtilis str. 168; CarEW, carboxylesterase from *B*. *subtilis* K91; EST1_THEFU, carboxylesterase from *Thermobifida fusca*; EST2A_MOUSE, pyrethroid hydrolase Ces2e from *Mus musculus*; EST1_MESAU, liver carboxylesterase from *Mesocricetus auratus*; and EST5A_FELCA, carboxylesterase 5A from *Felis catus*. Conserved motifs are highlighted. Residues strictly conserved among groups are shown in white font with red background. The possible catalytic triad (serine (S), glutamic acid (E), histidine (H)) is shown at the bottom of the alignment in green font. A conserved pentapeptide (GXSXG), containing the serine residue of the catalytic triad, was framed by a dotted box. Symbols above blocks of sequences represent the secondary structure, springs represent helices, and arrows represent β-strands.

A thermophilic *Bacillus* strain K91 growing in the range of 50–70°C with 50°C as the optimum growth temperature was isolated from a hot spring in Teng Chong, China. A comparison of the partial 16S rRNA with that deposited in the GenBank database showed 100% nucleotide identities to *B*. *subtilis* A3 type strain (accession no. GU301908.1), indicating that the two strains belong to the same genus. A gene annotated as “carboxylesterase” with a 1,464-long ORF that encoded 487 amino acid proteins was found and we named it as *CarEW*. No signal sequence was found. Sequence alignment revealed that amino acid sequence of CarEW were partially identical with some carboxylesterases from mammals, such as 37% sequence identity to EST2_HUMAN from *Homo sapiens* (accession: O0078), 37% to EST5A_FELCA from *Felis catus* (accession: Q8I034), 36% to SASB_ANAPL from *Anas platyrhynchos* (accession: Q04791) and 36% to EST2E_MOUSE from *Mus musculus* (accession: Q8BK48) (Fig. 1). Although the genes *EST5A_FELCA*, *SASB_ANAPL* and *EST2_HUMAN* have been cloned and characterized, there are no reports about their relevant function in the biodegradation of DiBP [38,39,40]. In addition, these sequences all contain the typical catalytic triad composed of Ser189–Glu289–His389 and the consensus motif (Gly-X-Ser-X-Gly) around the active-site serine (Fig. 1). The sequence similarities and conservation of typical catalytic triads suggest that these enzymes may have a number of important common functions that were conserved during the course of evolution.

**Fig 2:**
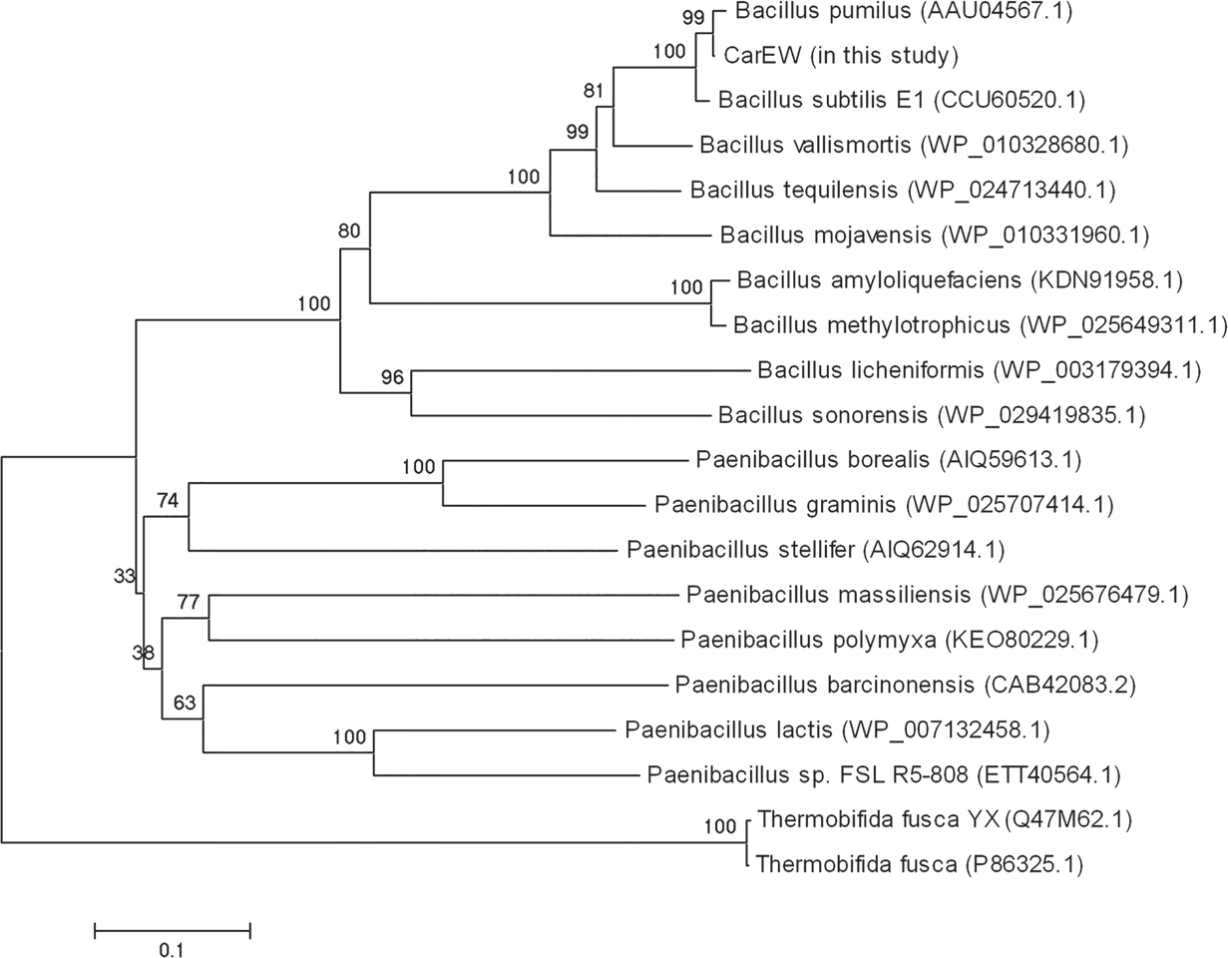
Neighbor-joining tree of esterases. Protein sequences were aligned using the built-in CLUSTAL W (default parameters), the tree was built using the neighbor-joining method with default parameters and 1000 bootstrap replications.

In addition, the deduced amino acid sequence of *CarEW* gene showed significant identity with sequences of other *Bacillus* and *Paenibacillus* species in the database. The *CarEW* shared 99% sequence identity to a carboxylesterase of *Bacillus pumilus* (AAU04567.1) whose function is unknown [41]. Phylogenetic analysis indicated that CarEW and carboxylesterase from *B*. *pumilus* clustered together, indicating the two enzymes came from the same genus strain and both belonged to the carboxylesterase family (Fig. 2). However, there are no reports on esterases from the *Bacillus* genus that can degrade DiBP.

### Enzyme cloning, overexpression and purification

**Fig 3:**
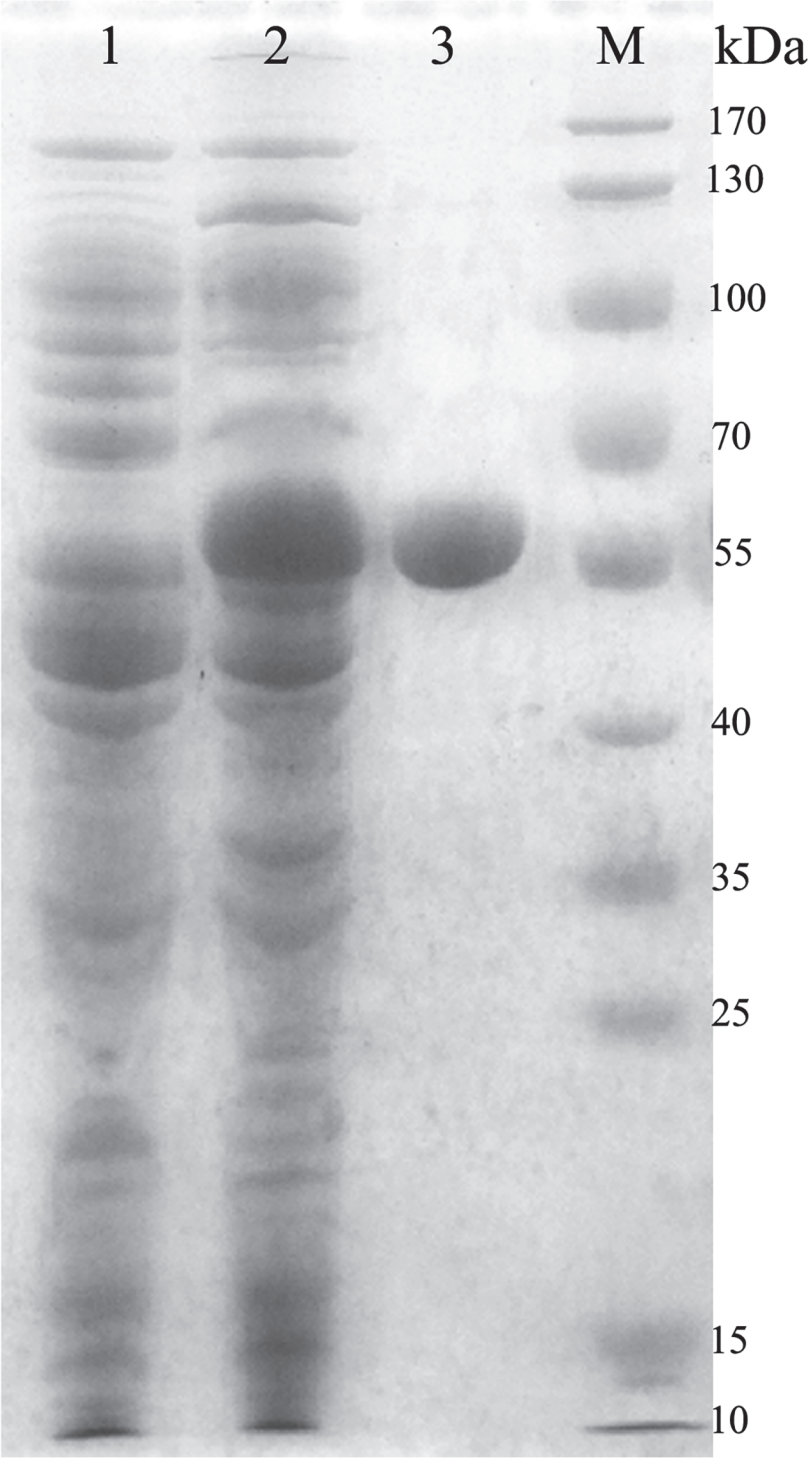
Analysis of the protein expressed in *E*. *coli* BL21 cells following purification on a 12% SDS-PAGE. Lane M, protein molecular marker; Lane 1, before induction with 0.7 mM IPTG; Lane 2, after induction with IPTG and grown at 20°C for 20 h; and Lane 3, purified recombinant CarEW (∼53.76 kDa).

To investigate the biochemical properties of the enzyme, the *CarEW* gene was expressed in the *pEASY*-E2 vector as a 6 × His tagged fusion protein and induced with 0.7 mM IPTG at 20°C for 20 h. The crude enzyme extracted from recombinant *E*. *coli* BL21 cells was purified using Ni^2+^–NTA metal–chelating affinity chromatography and analyzed by SDS–PAGE. As shown in Fig. 3 (lane 3), one band corresponded in size to the calculated molecular mass of CarEW was detected (∼53.76 kDa). The band was absent in the control lane from the *E*. *coli* BL21 cells carrying only the non-recombinant *pEASY*-E2 vector (Fig. 3, lane 1), which was cultured and induced under the same conditions as that for the *E*. *coli* BL21 (*pEASY*-E2-*CarEW*) cells. The isoelectric point (pI) was 4.88. The kinetic parameters of different substrates were determined at pH 7.5 and 45°C using the purified recombinant CarEW. ρ-NPC_4_ was the best substrate for CarEW according to the highest maximum initial velocity *K*_cat_/*K*_m_ values (Table 1). Besides, CarEW also displayed quite high enzyme activity towards ρ-NPC_2_, ρ-NPC_6_, ρ-NPC_8_, ρ-NPC_10_ according to the initial velocity *K*_cat_/*K*_m_ values in Table 1. The catalytic efficiency toward ρ-NPC_4_ was approximately 1.5-fold higher than toward ρ-NPC_2_. No significant esterase activity was observed for the substrates with a chain length ≥ C_12_. Compared with the ρ-NP substrates, the catalytic efficiency of DiBP and MiBP were not high. The *K*_cat_/*K*_m_ values for DiBP and MiBP were 0.109 and 0.031 (s^−1^ mM^−1^), respectively (Table 1).

**Table 1:** Kinetic parameters of the recombinant CarEW on different substrates at pH 7.5 and 45°C.

Substrate	*K*_m_ (mM)	*K*_cat_ (s^−1^)	*K*_cat_/*K*_m_ (s^−1^ mM^−1^)
*p*-NPC_2_	0.40±0.05	597.33±57.03	1493.33
*p*-NPC_4_	0.8±0.17	1792±172.43	2240
*p*-NPC_6_	0.69±0.09	689.23±57.03	998.88
*p*-NPC_8_	0.26±0.01	117.89±8.22	453.42
*p*-NPC_10_	0.21±0.04	86.15±0.48	410.24
*p*-NPC_12_	0.44±0.05	35.43±1.55	80.52
DiBP	1615.31±154.50	165.92±15.84	0.109
MiBP	76.37±0.05	2.25±0.02	0.031

Reactions were conducted in triplicates in 50 mM citrate phosphate buffer, pH 7.5, at 45°C, using different substrates over a concentration range of 0.1 to 1.2 mM.

### Effect of pH and temperature on enzyme activity and stability

**Fig 4:**
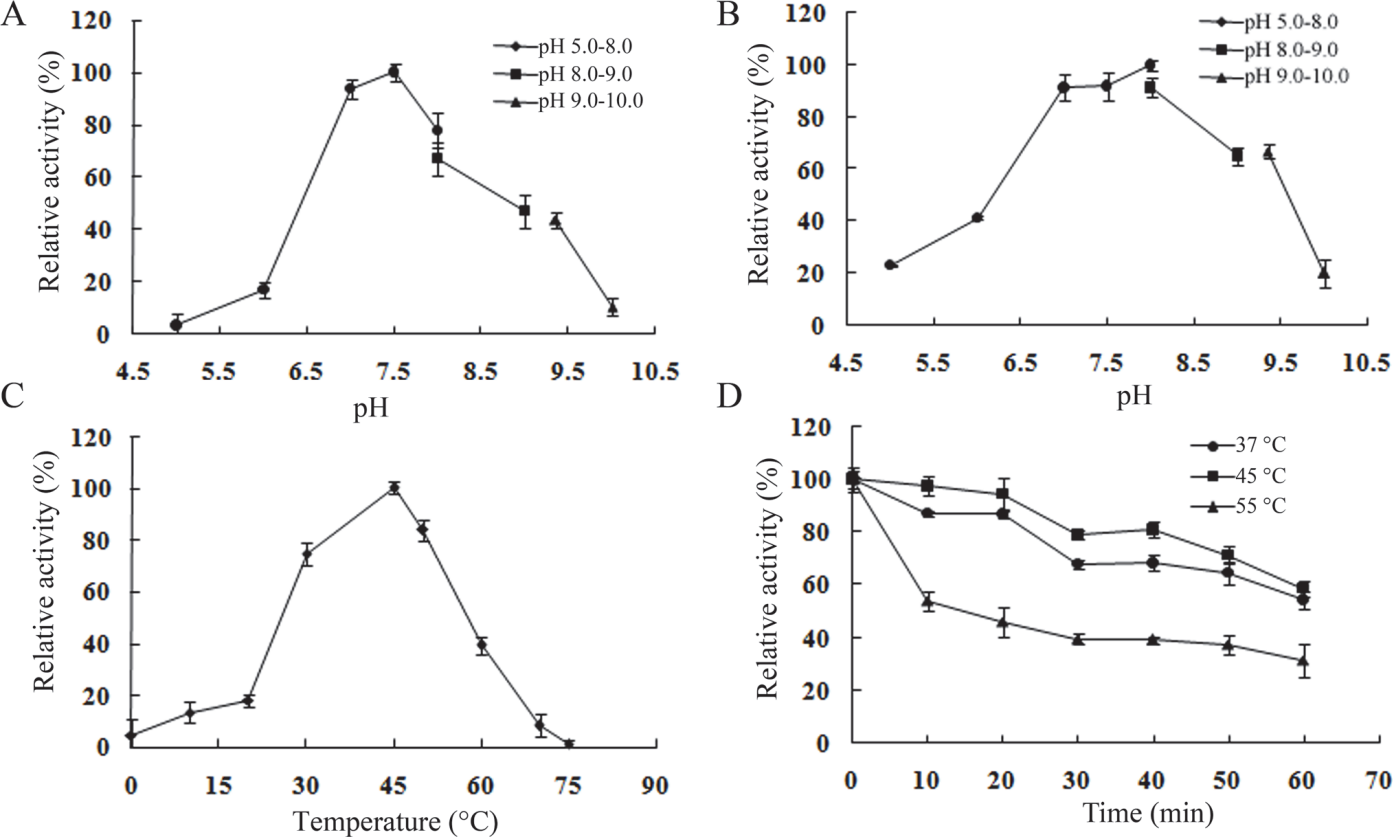
Effect of temperature and pH on CarEW activity and stability. Relative activity of purified CarEW was determined at different temperatures (A) or pH (C) using ρ-NP butyrate (ρ-NPC_4_) as the substrate at 405 nm. Remaining enzyme activity was measured at 45°C and pH 7.5 after incubating purified CarEW at different temperatures (B) or pH for 60 min. Error bars represent the mean±standard deviation (*n* = 3).

The effect of pH on CarEW activity was determined using ρ-NPC_4_ as the substrate at 45°C with pH values ranging from 5.0 to 10.0. Maximal activity was observed at pH 7.5. The enzyme activity was below 10% of the maximum activity when the pH was lower than 5.0 or higher than 10.0 (Fig. 4A). The pH stability analysis revealed that the enzyme was very stable at pH 6.5 to 9.5, retaining more than 60% of the original activity after pre-incubation at the given pH range for 1 h. However, CarEW only maintained 23% and 19% of its activity at pH 5.0 and 10.0, respectively, after incubation for 1 h (Fig. 4B).

The effect of temperature on CarEW activity was investigated using ρ-NPC_4_ as the substrate at pH 7.5 with the temperature ranging from 0°C to 75°C. Catalytic activity increased as temperature increased up to 45°C and then decreased beyond that level. No activity was observed when the temperature was higher than 75°C. CarEW displayed more than 60% catalytic activity at temperatures between 30°C and 50°C with an optimal temperature at 45°C (Fig. 4C), indicating that CarEW is a mesophilic enzyme. The temperature stability of CarEW was examined by measuring its residual activity after incubating the purified enzyme for 1 h at 37, 45, and 55°C. CarEW retained approximately 65%, 60%, and 40% of its activity after incubation for 1 h at 37, 45 and 55°C, respectively (Fig. 4D). The optimal temperature of 45°C and the optimal pH of 7.5 of CarEW activity in this study were similar to that from *Gordonia* sp. isolated from machine oil-contaminated soil [25]. CarEW was stable below 45°C, and hydrolase from *Gordonia* sp. was stable below 40°C. CarEW remained stable between pH 6.5 and 9.5, and this pH range of enzyme stability is broader than that for the hydrolase from *Gordonia* sp. (stable at pH 6.0–9.0). Moreover, the molecular size of the hydrolase from *Gordonia* sp. (31 kDa) was slightly different from CarEW (53.76 kDa) in our study, and the ability to degrade DiBP was not reported in their study [25]. The temperature and pH stability suggests that CarEW has the potential to be applied in the natural environment.

### Effect of metal ions, chemical agents and organic solvents on enzyme activity

The effect of different metal ions on CarEW activity was examined by addition of each metal ion into the reaction mixture at a final concentration of 1.0 mM. The results are presented in Table 2. Fe^2+^, Hg^2+^, Ag^+^, and Mn^2+^ had a strong inhibitory effect (50%–60% inhibition); Li^+^ and Co^2+^ had no apparent effect on enzyme activity (data not shown); whereas K^+^, Cu^2+^, Na^+^, and Zn^2+^ activated CarEW. CarEW activity was moderately inhibited by Ca^2+^, Ni^2+^, Ba^2+^, and Mg^2+^ (17%–30% inhibition). Chemical agents such as CTAB (0.1%) strongly inhibited CarEW (7.8% residual activity), and inhibition by DTT (1 mM), Triton X-100 (2%) and SDS (0.1%) were approximately 63%, 48%, and 40%, respectively. Urea (0.1 M) and EDTA (1 mM) exhibited moderate inhibitory effects (residual activities from 77% to 85%). Tween 80 had little effect on the enzyme activity.

**Table 2:** Effect of various metal ions and chemical agents on enzyme activity.

Metal ions/Chemical agents	Concentration	Relative activity (%)
Cu^2+^	1 mM	122.1±2.3
K^+^	1 mM	121.4±1.2
Na^+^	1 mM	113.1±2.3
Zn^2+^	1 mM	101.2±0.5
Al^3+^	1 mM	90.2±2.5
Mg^2+^	1 mM	89.6±2.9
Ba^2+^	1 mM	83.1±1.1
Ca^2+^	1 mM	74.8±2.3
Ni^2+^	1 mM	72.6±3.7
Ag^+^	1 mM	40.4±12.3
Fe^2+^	1 mM	32.4±8.1
Mn^2+^	1 mM	26.4±12.7
Hg^2+^	1 mM	25.4±3.2
SDS	0.10%	60.3±3.7
CTAB	0.10%	7.8±5.9
Tween 80	2%	94.5±3.0
Triton X-100	2%	52.6±3.5
Urea	0.1 M	76.9±1.1
DTT	1 mM	37.2±7.3
EDTA	1 mM	85.5±2.1

The enzyme was incubated with each metal ion or chemical agent at 45°C for 5 min, and residual activities of CarEW were measured using ρ-NPC_4_ as the substrate. The activity of the enzyme not incubated with metal ions or chemical agents was defined as 100%. The data is represented as an average of three replicates.

Investigation on the tolerance of any enzyme to organic solvents is critical for industrial applications. CarEW stability in different organic solvents was determined by incubation with methanol, ethanol, acetone, 2-propanol, DMSO^b^, N-propanol, or methyl cyanide at the indicated concentrations. Residual activity of the treated enzymes was then determined under the standard assay condition. As shown in Table 3, acetone (2%), DMSO^b^ (2%), and N-propanol (2%) had little effect on the enzyme activity (81%, 81%, 78% residual activities, respectively), whereas methanol, ethanol, and 2-propanol (2%) strongly activated CarEW with residual activities of 114%, 117%, and 149%, respectively. Methyl cyanide had little effect on CarEW activity. Majority of the organic solvents tested in this experiment are used extensively in industrial applications and consumer products, and they are inevitably released to the environment during their production, storage, handling, distribution, and use [42]. Therefore, these organic solvents could coexist with the ubiquitous pollutants PAEs in the environment. Therefore, a PAE-degrading enzyme must maintain stability even in the presence of these chemicals. The stabilities of CarEW in the presence of organic solvents and a number of the chemical agents are also critical in PAE-degradation reactions in complex environments.

**Table 3:** Effect of organic solvents on enzyme stability after incubation at 45°C for 1 h.

Organic solvents	concentration	Relative activity (%)
Methanol	2%	114.7±3.7
Ethanol	2%	117.9±0.7
Acetone	2%	81.5±0.9
2-Propanol	2%	149.4±0.7
DMSO^b^	2%	81.6±4.7
N-propanol	2%	78.6±6.0
Methyl cyanide	2%	92.7±2.5

No organic solvent was included in the control samples. The concentrations of organic solvents are indicated (v/v). The enzyme activity was determined under the standard assay condition, and the data is represented as an average of three replicates.

### Identification of intermediate products and the probable pathway of DiBP degradation

**Fig 5:**
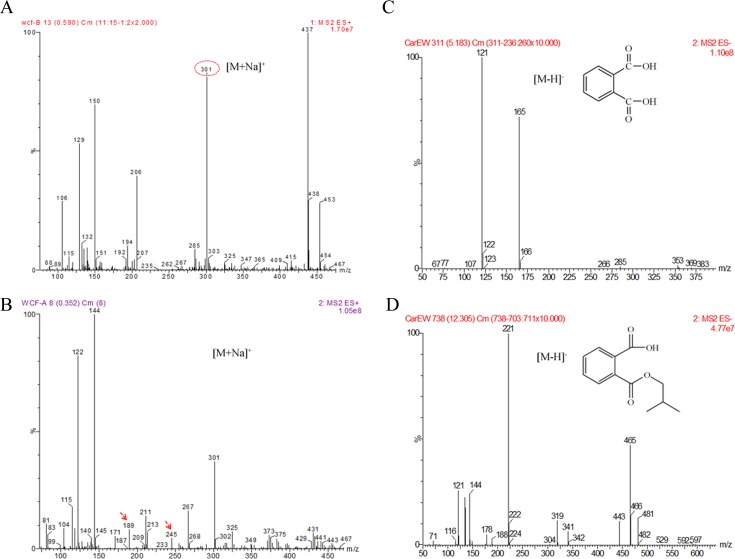
ESI-MS analysis of products identified in the CarEW catalyzed reaction. (A) and (B) The full ion scan of DiBP degradation after incubation with CarEW and the control without CarEW. m/z 301, DiBP; DiBP incubation with CarEW. m/z 189, PTH; m/z 245, MiBP. (A) and (B) were both tested under the positive mode ([M+Na]^+^). (C) and (D) Daughter ion scan for metabolites derived from the biodegradation of DiBP. (C) m/z 121, phthalate acid (PTH); (D) m/z 221, monoisobutyl phthalate (MiBP). Both were tested under negative mode ([M-H]^−^).

**Fig 6:**
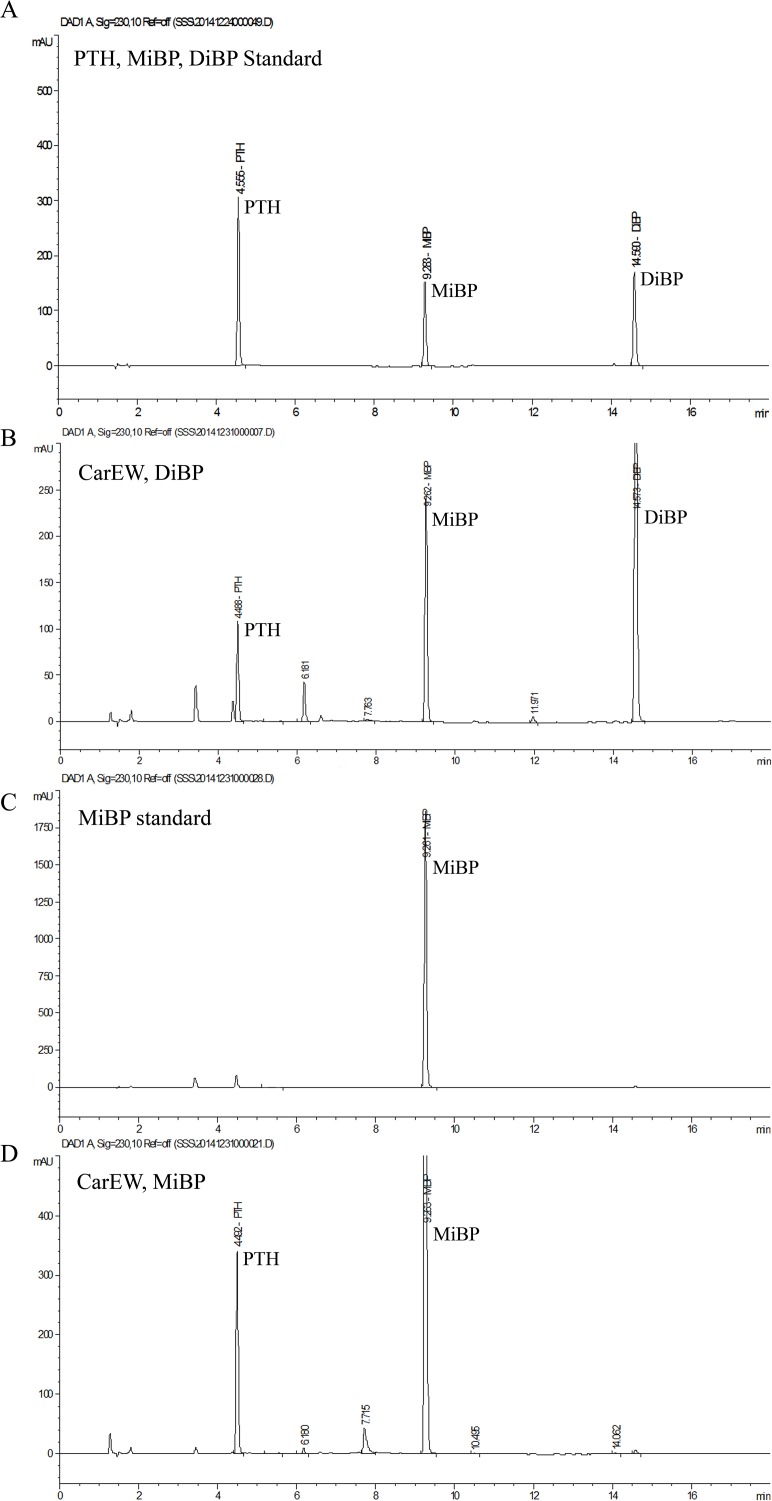
The results of HPLC analyses of catabolic intermediates. (A) DiBP, MiBP and PTH mixture for the standard sample. (B) The results of DiBP degradation after incubation with CarEW. (C) Reaction of MiBP with 10 mM citric acid-Na_2_HPO_4_ solution (pH 7.5) buffer instead of CarEW. (D) The results of MiBP degradation after incubation with CarEW.

**Fig 7:**
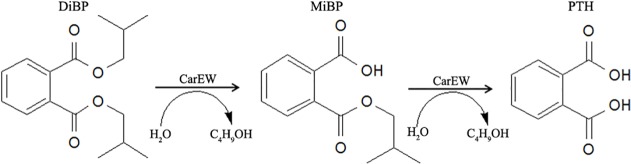
Proposed pathway for incomplete diisobutyl phthalate degradation by CarEW.

The products of the reaction catalyzed by CarEW were identified by comparison to the mass spectra (m/z) of reaction with no enzyme using ESI-MS. Firstly, a full ion scan using the DiBP mixture without the enzyme CarEW as the control (Fig. 5) was performed. In Fig. 5B, we detected DiBP (m/z 301) and two other possible products PTH and MiBP which exhibited [M+Na]^+^ molecule m/z of 189 and 245. However, only DiBP (m/z 301) was detected in the control in Fig. 5A. To further confirm the two possible products were PTH and MiBP, a daughter ion scan (ESI-MS-MS) was used. Two corresponded products phthalic acid (PTH; m/z 165) and its mono-derivative monoisobutyl phthalate (MiBP; m/z 221) were identified. The ion polarities of the two compounds were both negative ([M-H]^¯^) (Fig. 5C and D). On the basis of the potential biases for detecting compounds among assays, the main intermediate products were further identified by HPLC using the mixture of DiBP, MiBP, and PTH for the standard (Fig. 6A). The PTH and MiBP were identified in the reaction of DiBP with the enzyme CarEW at the retention time 4.488 and 9.262, which were close to the standard sample mixture (Fig. 6B). In order to further test whether MiBP can be hydrolyzed to PTH by CarEW, the reaction of MiBP with CarEW was performed used the same conditions as DiBP. The result showed that MiBP can also be hydrolyzed to PTH by CarEW (Fig. 6D). No PTH was detected in the MiBP reaction without the enzyme CarEW (Fig. 6C). The combined results of ESI-MS and HPLC led to the identification of two products, namely, PTH and MiBP. And the main degradation process of DiBP is supposed to be the stepwise hydrolysis of the ester bonds. On the basis of the products, a pathway for incomplete DiBP degradation by CarEW was proposed (Fig. 7).

Therefore, the greatest value of CarEW was its activity to both of the carboxylic ester linkages. Most of the esterases identified at present can only hydrolyze one of the ester bonds. The esterase from *Fusarium* sp. DMT53 can only hydrolyze the first carboxylic ester linkage of DMT to monomethyl terephthalate (MMT) but lack the ability to remove the second linkage [23]. Two distinct esterases from *Micrococcus* sp. YGJ1 were involved in the metabolism of dialkyl phthalates (DAPs), including DAP esterase and monoalkyl phthalate (MAP) esterase [15,24]. DAP esterase was responsible for the hydrolysis of DAP to MAP that was further transformed to phthalic acid by the action of MAP esterase. Hydrolase PatE from *R*. *jostii* RHA1 specifically hydrolyzed monoalkyl PAEs to phthalic acid but did not transform dialkyl PAEs [26]. Esterase dphB from a metagenomic library specially catalyzed the hydrolysis of dipropyl phthalate, dibutyl phthalate, and dipentyl phthalate to the corresponding monoalkyl PAEs [27]. A newly identified esterase Est1 from *S*. *acidophilus* DSM10332 degraded PAEs to their corresponding monoalkyl PAEs [29]. These observations and our results suggested that phthalate esterases are a diverse group of distinct enzymes involved in the cleavage of the carboxylic ester linkages of PAEs.

There are no reports on the biodegradation of DiBP by microorganisms or their enzymes to date. To the best of our knowledge, we are the first to report that DiBP can be degraded by an esterase, and we will investigate the biodegradation of other kinds of PAEs using CarEW in future. Furthermore, *Bacillus* sp. K91 is a widely distributed nonpathogenic bacterium and has the ability to degrade DiBP or other kinds of PAEs. Thus, if more genes or enzymes, as well as pathways, involved in PAEs biodegradation were characterized, this strain could be a candidate host for the design of a very useful “super bacterium” for degrading recalcitrant chemicals especially the commonly used PAEs.

## Conclusions

In this study, a newly identified and promising carboxylesterase gene, *CarEW*, that displays very high catalytic activity towards ρ-NP substrates and positive stability under broad temperature, wide pH range, and organic solvents, is cloned and characterized. Moreover, CarEW is capable of hydrolyzing DiBP to MiBP and PTH. CarEW can completely degrade DiBP to PTH over a long duration under ambient temperatures. To our knowledge, this is the first report on a carboxylesterase enzyme that catalyzes the hydrolysis of DiBP to PTH, which we propose is important for the bioremediation of widespread environmental pollution caused by plasticizers. In the future, more genes, enzymes, or other microorganisms involved in the complete mineralization of DiBP or other PAEs should be studied.

## Supporting Information

S1 Table Identities between amino acid sequence of CarEW and those of other reported phthalate esterases or hydrolases. (PDF)
