# Supplementary material for: Properties of a Newly Identified Esterase from Bacillus sp. K91 and Its Novel Function in Diisobutyl Phthalate Degradation
Source: PLoS One. 2015 Mar 6;10(3):e0119216. doi: 10.1371/journal.pone.0119216 (PMC4352063; doi:10.1371/journal.pone.0119216)
Supplement: S1 Table — (PDF) [file pone.0119216.s001.pdf]

S1 Table. Identities between amino acid sequence of CarEW and those of other reported phthalate esterases or hydrolases.

Enzyme	Source of enzyme	Phthalate substrate	Identities with CarEW	Query cover	E-value	Reference
Esterase	*Fusarium* sp. DMT-5-3	DMT	–[a]	–	–	23
Esterase	*Micrococcus* sp.YGJ1	PAEs	–[a]	–	–	24
MEHP hydrolase	*Gordonia* sp. Strain P8219	PAEs	38%	10%	0.017	25
PatE	*Rhodococcus jostii* RHA1	PAEs	38%	17%	1.3	26
DphB	Metagenomic library	PAEs	33%	68%	5e-08	27
DBP hydrolase	*Acinetobacter* sp. Strain M673	PAEs	28%	42%	2e-08	28
esterase EstS1	*Sulfobacillus acidophilus*	PAEs	21%	62%	0.003	29
Cholesterol esterases	Porcine and bovine pancreases	PAEs	–[a]	–	–	30
Cutinase	*F. oxysporum* f. sp. pisi	PAEs	–[a]	–	–	31

–[a], no sequence available;

The abbreviations used are: MEHP hydrolase: Mono-2-Ethylhexyl Phthalate Hydrolase; PatE: phthalate ester hydrolase; DphB: phthalate esters hydrolase; DBP: dibutyl phthalate;
